# Mechanism of mesenchymal stem cells and exosomes in the treatment of age-related diseases

**DOI:** 10.3389/fimmu.2023.1181308

**Published:** 2023-05-18

**Authors:** Jia Li, Yuling Huang, Haiyan Sun, Lina Yang

**Affiliations:** ^1^ Departments of Geriatrics, The First Hospital of China Medical University, Shenyang, China; ^2^ Department of Endodontics, School of Stomatology, China Medical University, Shenyang, China

**Keywords:** age-related diseases, exosomes, mesenchymal stem cells, aging, cell signaling

## Abstract

Mesenchymal stem cells (MSCs) from multiple tissues have the capability of multidirectional differentiation and self-renewal. Many reports indicated that MSCs exert curative effects on a variety of age-related diseases through regeneration and repair of aging cells and organs. However, as research has progressed, it has become clear that it is the MSCs derived exosomes (MSC-Exos) that may have a real role to play, and that they can be modified to achieve better therapeutic results, making them even more advantageous than MSCs for treating disease. This review generalizes the biological characteristics of MSCs and exosomes and their mechanisms in treating age-related diseases, for example, MSCs and their exosomes can treat age-related diseases through mechanisms such as oxidative stress (OS), Wnt/β-catenin signaling pathway, mitogen-activated protein kinases (MAPK) signaling pathway, and so on. In addition, current *in vivo* and *in vitro* trials are described, and ongoing clinical trials are discussed, as well as the prospects and challenges for the future use of exosomes in disease treatment. This review will provide references for using exosomes to treat age-related diseases.

## 1 Introduction

The global average life span has increased in recent years, with the number of people over 60 years of age rising to 22% between 2000 and 2050, indicating that aging remains a serious problem (1). Aging is seen as a huge socio-economic challenge that most countries will face in the coming decades. Cellular aging is a state of replication stagnation in cells and is also an important marker of aging, closely related to aging-related diseases. With age, the accumulation of aging cells accelerates the aging process of the body (2). Aging causes gradual loss of function in tissues and organs. This is a natural, inevitable, physiological phenomenon, which causes the degradation of various physiological functions and weakens the ability to repair and regenerate (3). As research deepens, people regard preventing or reducing aging cells as an important means of mitigating aging (4). Many studies have found that MSCs can be effective in repairing and regenerating cells and have therapeutic effects on age-related diseases. However, due to its intrinsic characteristics and the limitations of transplantation technology, the current trend is to develop and study various functions of exosomes. For example, research has shown that as one of the methods for treating osteoarthritis (OA), exosomes can act as carriers to deliver microRNA-140 specifically into chondrocytes for therapeutic effects (5). MSC-Exos combined with curcumin reach target cells and relieve Parkinson’s disease (PD) progression through a variety of functions, such as increasing drug concentration, reducing inflammation response, and promoting neuronal repair (6). In addition, in the myocardial ischemia-reperfusion (I/R) model, MSC-Exos have immunomodulatory effects on macrophages mainly by inhibiting Toll-like receptor 4 activity through the transfer of exosomal miR-182, thereby alleviating myocardial injury (7). Finally, Xia et al. used the rabbit intervertebral disc degeneration (IVDD) model to discover that MSC-Exos can improve the degenerative changes of IVDD by suppressing inflammation and supplementing mitochondrial proteins (8). The study of exosomes is not only limited to cellular or animal experiments but many related clinical trials have also been conducted, which provides assistance for the future clinical application of exosomes.

## 2 MSCs and exosomes

### 2.1 Biological characteristics of MSCs

Commonly, stem cells could be classified into adult stem cells such as MSCs, induced pluripotent stem cells (iPSCs), embryonic stem cells (ESCs), and other types of stem cells (9). ESCs are stem cells that can proliferate indefinitely and differentiate into almost all cell lines without showing senescence (10, 11). The complex ethics of stem cells make their use even more subject to caution. MSCs can be found in nearly all tissues, such as bone marrow derived MSCs (BMSCs), adipose-derived MSCs (ADMSCs), human umbilical cord-derived MSCs (huMSCs), human placenta-derived MSCs (hpMSCs), and MSCs in other tissues (11). At present, research has found that some mechanisms of MSCs therapy for diseases include 1) secretion of proteins/peptides and hormones; 2) transfer mitochondria in a variety of ways; and 3) transfer microvesicles or microcapsules containing RNA, protein, and other substances (12). Many studies have proved that MSCs are also widely used to cure age-related diseases. For instance, MSCs can reduce skin contraction and improve appearance when treating skin wounds (13), having the potential to ameliorate myocardial infarction in cardiovascular (14), MSCs also can alleviate the degeneration of articular cartilage (15) and improve a variety of neurological diseases (16). Although the mechanisms of aging are complex, MSCs attenuate aging through a variety of pathways. The mechanisms of MSCs and exosomes in different diseases in this review are displayed in **Table 1**.

**Table 1:** MSCs and their exosomes treat age-related diseases through a variety of mechanisms.

Mechanism	Disease	Model	Animal	Source of MSCs/Exos/EVs	Effect	Ref.
Oxidative stress	Skin injury	H_2_O_2_-stimulated epidermal keratinocytes and UV-irradiated wild type	Mice	MSC-Exos	Through the NRF2 signaling pathway to reduce the generation of ROS	(17)
	Skin injury	I/R	Mice	MSCs	Reducing ROS generation and apoptosis	(18)
	AD	Vivo AD model	Rat	MSCs and MSC-EVs	Reducing AβO-induced oxidative stress and synapse damage	(19)
	Atherosclerosis	Endothelial cells isolated from human umbilical cord veins were cultured in the presence of H_2_O_2_ and monocytes		DBMSCs	Reducing oxidative stress and immune cells induced injury	(20)
	IVDD	IVDD model	rabbit	MSC-Exos	Inhibit the development of inflammation by inhibiting the activation of the NLRP3 inflammasome	(8)
	COPD	Ozone-exposed mice and ASMCs were cultured in the presence of cigarette smoke medium	Mouse	iPSC-MSCs	Reducing oxidative stress-induced mitochondrial dysfunction	(21)
Wnt/β-catenin Signaling Pathwy	SCI	SCI model	Wistar rat	BMSC-Exos	Reducing tissue damage and neural cell apoptosis, and promoting functional recovery	(22)
	PD	MPTP/6OHDA mouse model	Mice	HSPCs	Inhibiting secretion of pro-inflammatory cytokines and neural cell apoptosis	(23)
	AD	AD rats and neurons	Rat	BMSC-EVs	Delivering miR-29c-3p to neurons to inhibit BACE1 expression	(24)
	Cerebral ischemia/reperfusion injury	MCAO model	Male Wistar rat	hAMSCs	Restoring endogenous antioxidant system and suppressing apoptotic cell death through FoxO1 and Wnt/β-catenin signaling pathway	(25)
	Ischemic myocardium I/R injury	Myocardial I/R model	Male SD rat	ADMSC-Exos	Reducing I/R-induced necrosis,apoptosis and hypoxia/reoxygenation-induced injury	(26)
	Cutaneous wound	Rat skin burn model	Rat	hucMSC-Exos	Delivering Wnt4 to activate the Wnt/β-catenin signaling pathway	(27)
	Cutaneous wound	Skin lesion model		ADMSC-Exos	Promoting cell proliferation and migration of HaCaT cells, and repressing cell apoptosis of HaCaT cells	(28)
MAPK signaling pathway	Cerebral ischemic injury	MCAO model	Adult male Sprague–Dawley rat	hDPSCs	Increasing neuroprotective and anti-inflammatory molecules, resulting in the neuronal rescue and survival	(29)
	ONFH	ONFH rabbit model	Mature New Zealand rabbit	BMSC-Exos	BMSC-Exos carrying over-expressed miR-122-5p attenuated ONFH development	(30)
	Osteoarthritis	BMSCs were cultured with TGF-β1		BMSCs	Suppressing the p38 pathway, the chondrogenesis can be inhibited	(31)
	COPD	The rats were exposed to cigarette smoke	Rat	MSCs	Suppressing the p38 MAPK and ERK pathway, down-regulating COX-2/PGE2 in macrophages	(32)
	Atherosclerosis	Low-density lipoprotein receptor-deficient mice were fed a high-fat diet	Five- to six-week-old male mice	MSC-EVs	Suppressing MAPK signaling pathway, CAM expression, and macrophage accumulation in the vascular walls	(33)
Sirtuin	Myocardial infarction	MI rat model	Rat	hMSC-Exos	The ncRNA KLF3-AS1 in hMSC-Exos can inhibit cardiomyocyte activity	(34)
	Damaged cartilage, or in inflammatory joint arthritis	CIA mouse model	Mice	MSCs	Suppressing T helper (Th)-17 cell activation and increasing the Treg cell population	(35)
	UV radiation-induced skin photodamage and aging	Rat model of acute skin photodamage	Rat	hucMSC-Exos	hucMSC-Exos can promote the expression of SIRT1 in HaCaT cells by transporting 14-3-3ζ protein	(36)

MSC-Exos, exosomes derived from MSCs; ROS, reactive oxygen species; I/R, ischemia-reperfusion; MSCs, mesenchymal stem cells; AD, Alzheimer’s disease; MSC EVs, mesenchymal stem cell-derived extracellular vesicles; PD, Parkinson’s disease; DBMSCs, decidua basalis mesenchymal stem/multipotent stromal cells; IVDD, intervertebral disc degeneration; ASMCs, airway smooth muscle cells; iPSC-MSCs, induced-pluripotent stem cell-derived MSCs; COPD, chronic obstructive pulmonary disease; SCI, spinal cord injury; BM-MSC-EVs, bone marrow MSC-EVs; BMSC-Exos, exosomes derived from BMSCs; hAMSCs, human amniotic mesenchymal stem cells; MCAO, middle cerebral artery occlusion; ADMSC-Exos, exosomes derived from ADMSCs; hucMSC-Exos, human umbilical cord mesenchymal stem cells; ADMSCs, adipose-derived MSCs; STZ, streptozotocin; hDPSCs, human dental pulp stem cells; ONFH, osteonecrosis of the femoral head; BMSCs, bone marrow-derived mesenchymal stem cells; BMSC-Exos, exosomes derived from BMSCs; MI, myocardial infarction; UV, ultraviolet; CIA, collagen-induced arthritis.

### 2.2 Function of MSCs

Many data support the view that MSCs play a role in a paracrine way. Studies proved that MSC-Exos have functions akin to MSCs, such as repairing tissue damage, inhibiting inflammatory response, and regulating the immune system. They also exhibit antioxidant and anti-apoptotic abilities. For example, transplantation of human MSCs into diabetic mice promoted healing by aggregating large numbers of MSCs to the wound site and producing growth factors such as platelet-derived growth factor (PDGF) receptor-α and vascular endothelial growth factor (VEGF). Endothelial cells can promote angiogenesis and express various cytokines and growth factors that contribute to wound repair, involving transforming growth factor (TGF)-β, interleukin-1 (IL-1), IL-5, and IL-6, which are beneficial to wound repair (13). Research has shown that BMSCs have a positive effect on the damaged microenvironment and promote tissue regeneration by releasing bioactive factors (37). Chen et al. (38) discovered that astrocytes cocultured with BMSCs decreased astrocytes apoptosis by down-regulating pro-inflammatory factors and up-regulating anti-inflammatory factors. The paracrine effect of MSCs is to mediate the communication between surrounding cells through the production of growth factors, cytokines, and other regulatory molecules so as to inhibit immune response and improve antioxidant capacity (39). Many substances are found in the MSC-derived secretome (40). The extracellular vesicles derived from MSCs (MSC EVs) contain mRNA, cytokines, microRNAs, immunomodulatory factors, chemokines, and so on. These molecules can modulate the function, phenotype, and homing of immune cells (41). There are various types of extracellular vesicles, which can be classified according to their diameter size. Small extracellular vesicles include exosomes, small ectosomes, and so on, while microvesicles belong to medium-sized vesicles, and large vesicles include apoptotic bodies, large oncosomes, and so on (42).Each type has its own characteristics. The diameter of an exosome is between 30 and 150 nm, nevertheless, exosomes may differ in size depending on how they are isolated (43). The membranes of early endosomes bud inward to form exosomes, which eventually become multivesicular bodies (44). These are involved in cell-to-cell communication (45, 46). Microvesicles with sizes ranging from 50 to 1000 nm are derived from the outer buds of the plasma membrane (PM) of a cell but their formation is not well defined (47). Apoptotic bodies are formed when apoptotic cells fragment and a PM bleb forms, and their size ranges from 50 to 5000 nm (48). The stress caused by cell contraction separates the cytoskeleton from the membrane, and the apoptotic body forms (44).

### 2.3 Biogenesis of exosomes

**Figure 1:**
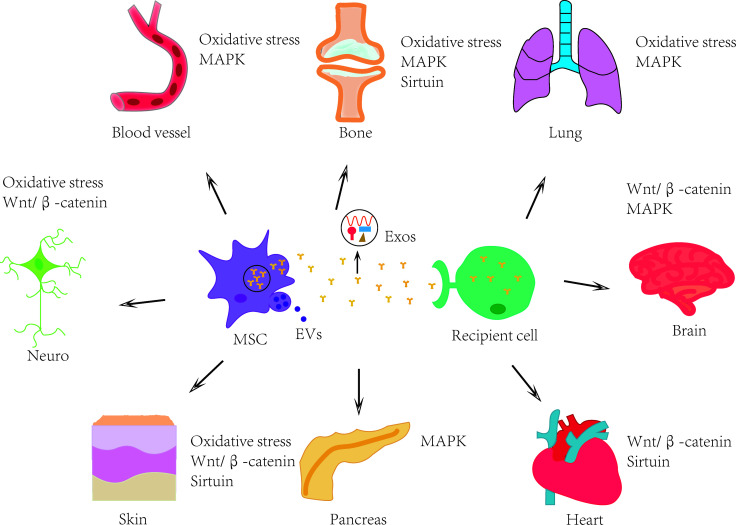
MSCs and their exosomes have therapeutic effects on multiple human systems and organs. MSCs and their exosomes can treat age-related diseases involving nervous, skin, skeletal, cardiovascular and other systems through mechanisms such as oxidative stress, Wnt/β-catenin signaling pathway, MAPK signaling pathway and sirtuin family. MSC: mesenchymal stem cell; EVs: extracellular vesicles; Exos: exosomes.

Exosomes are secreted by many different types of cells and are present in various physiological fluids (49), including amniotic fluid, blood, urine, saliva, and so on. When exosomes successfully enter the target cell, they could modulate the function and signal transduction thereof (45). The biogenesis of exosomes is closely related to the endosomal system and its transport pathway. Primary endocytic vesicles, early endosomes (EEs), and multivesicular bodies (MVBs) all belong to the endosomal system (50). The first stage is the formation of EEs. Proteins on the PM could be moved to the exterior of EEs. EEs are located at the cell membrane, where they can sort the recovered cargoes from the PM and then target the internal digestion recovery capsule. In the second step, the limiting membrane invaginates and forms intraluminal vesicles (ILVs). Since late endosomes could include multiple ILVs, they are also known as MVBs. MVBs fuse with the PM and release ILVs outside the cell to produce exosomes (51, 52). It is noteworthy that not all vesicles within the MVBs are released as exosomes; a portion of the MVBs can be carried into lysosomes for degradation and exosome fragments are recycled. Exosomes can make contact with target cells through endocytosis, and ligand-receptor direct or indirect binding (53). Other researchers also showed that MSC-Exos have a similar biological activity to MSCs, therefore it can replace MSCs to treat certain diseases (54, 55). Exosomes are more convenient and effective due to their high safety, and low immunogenicity (56). In short, they can work in a variety of ways, as shown in **Figure 1**.

### 2.4 The advantages of exosomes

Although MSCs have many functions, their therapeutic effects are controversial (57), and some problems may be unavoidable, such as infusion toxicity (58), cell rejection, the low survival rate *in vivo*, and low targeting ability; however, MSC-Exos not only have similar biological activities as MSCs but also show higher safety, low immunogenicity and do not directly form tumors (56). Firstly, due to the ease of obtaining and purification, and the high sensitivity and specificity, exosomes have become biological markers for various diseases. For example, plasma-derived exosomal miR-30e and miR-92a are expressed more in atherosclerosis and can serve as biomarkers for the diagnosis and treatment of atherosclerosis (59). In addition, exosomes can also serve as biomarkers for various diseases such as PD, fibrosis (60), and cancer (61). Secondly, exosomes are much smaller than MSCs so can avoid being engulfed by macrophages and pass through the microvascular system more smoothly (62). More importantly, exosomes possess a phospholipid bilayer structure and can also penetrate the blood-brain barrier, making them effective carriers for the delivery of proteins, genetic material, and various drugs to target cells (54). Finally, compared with pathway inhibitors, exosomes can act on different targets. While inhibiting this pathway, exosomes can also inhibit cell apoptosis, fibrosis, and inflammation, regulate immunity, and provide a repairing environment for damaged cells. In addition, exosomes can be developed and optimized through various methods, achieving better therapeutic effects by drug pre-treatment and gene or peptide modifications. Moreover, exosomes have multiple modes of action (63). They can not only play a role themselves, but also act as carriers to transport various substances to targets, with a wider range of effects, and can even cross capillaries and the blood-brain barrier (64). Overall, the application of exosomes has more advantages.

## 3 Mechanism of action of MSCs and exosomes in the treatment of age-related diseases

### 3.1 Oxidative stress

An imbalance between oxidant production and antioxidant defenses can lead to OS. This phenomenon increases with age and eventually affects the normal function of certain tissues. OS has been implicated in a variety of age-related diseases including atherosclerosis, Alzheimer’s disease (AD), chronic obstructive pulmonary disease (COPD), *etc.* (65). There are many ways in which MSCs and exosomes reduce OS, among which inhibiting inflammation is one of the most important. Research showed that ultraviolet (UV) irradiation can reduce the activity of antioxidant enzymes in skin cells or tissues (66). OS can accompany inflammation, which may also lead to subsequent oxidative damage (67). OS is one of the main causes of skin damage caused by various harmful stimuli such as UV radiation (68). Wang et al. (17) have demonstrated that co-culturing 5μg/ml of extracellular vesicles with H_2_O_2_-stimulated keratinocytes for 12 hours, and injecting 2μg of MSC-Exos into UV-irradiated mouse models daily for 5 consecutive days, resulted inreduced ROS production, abnormal calcium signal, DNA damage, and mitochondrial changes, thereby reducing inflammatory responses and alleviating cell and tissue responses induced by OS. These effects are mainly mediated by the NrF2 signaling pathway. Nrf2 is a transcription factor with numerous target genes, which can play a role through a variety of signal axes to reduce the damage caused by OS and inflammation. Nrf2 also plays a part in the anti-oxidative stress effect of MSCs and their exosomes. The positive effects of Nrf2-ARE Pathway in improving nerve function and alleviating nerve injury (69) and kidney injury (70) have been reported in kinds of literature. In addition, the signaling pathway Nrf2/HO-1 not only helps neurodegenerative diseases (71) but also alleviates myocardial infarction (MI) (72). In addition, I/R injury is one of the developmental mechanisms of pressure ulcers, and inhibiting OS can effectively alleviate I/R injury. Motegi et al. (18) proved that the injection of MSCs (2 × 10^6^ cells) in the mice I/R model could reduce the levels of Nrf2, Nox2, and HO-1 and promote the secretion of growth factors or cytokines such as VEGF, basic fibroblast growth factor, PDGF, and angiopoietin-1. It is suggested that the injection of MSCs may prevent the occurrence and development of pressure ulcers by reducing cellular and vascular damage, OS, and apoptosis.

As aging intensifies, it is estimated that in the future, 3% to 5% of the population aged 65 and above may suffer from AD (73). The abnormal accumulation of amyloid-β peptide oligomers (AβOs) is one of the causes of AD, and it also participates in neuronal OS (74). Godoy et al. (19) revealed that MSCs and MSC EVs inhibit neurocyte damage caused by AβOs through the following mechanisms:1) accelerating AβOs internalization and degradation; 2) release of EVs including antioxidant enzyme and catalase; 3) selective secretion of IL-10, VEGF, and IL-6. Cui et al. (75) discovered that the expressions of IL-6, IL-β, and TNF-α were remarkably down-regulated, while the expressions of IL-4, IL-13, and IL-10 were remarkably increased after injecting 5 × 10^11^ MSC-Exos into the brain tissue of AD mice. Diseases of cardiovascular and cerebrovascular aging, such as atherosclerosis, and OS results from the accumulation of low-density lipoprotein (LDL) and immune cells, thereby changing the functional activity of proteins, producing more toxic free radicals and causing endothelial cell damage (76). Concerning this aspect of the problem, Alshabibi et al. (20) stated that human decidua basalis mesenchymal stem/multipotent stromal cells (DBMSCs) can regulate some genes that mediate endothelial cell proliferation, permeability, and monocyte infiltration, and enhance glutathione and thioredoxin reductases activities. These results implied that DBMSCs can protect endothelial cells from OS and inflammation to treat inflammatory diseases such as atherosclerosis. Similarly, BMSCs derived exosome miR-181a-5p combined with ATF2 alleviates cardiomyocyte inflammation induced by OS (77).

Links between OS and other age-related diseases have also been reported in the literature, for instance, long-term OS and inflammation are important factors leading to the development of IVDD. OS has been linked to the pathological mechanism of IVDD in numerous studies (78). Xia et al. (8) injected 15μg MSC-Exos into the intervertebral disc of the rabbit IVDD model to estimate the interference effect of MSC-Exos on H_2_O_2_ production: in this process, the low levels of caspase-9 and caspase-3 indicated that MSC-Exos could downregulate the ROS level in NP cells and reduce NP cell apoptosis. MSC-Exos can inhibit the development of inflammation through inhibiting the activation of NLRP3, which may provide a reference for the treatment of IVDD (79). In addition, huMSCs derived exosome miR-100-5p protects chondrocytes by targeting NOX4, thus treating OA (80). Cigarette smoke is one of the sources of ROS, and sustained OS is an important factor leading to COPD. Li et al. (21) verified that iPSC derived MSCs (iPSC-MSCs) could ameliorate OS-induced mitochondrial function changes by decreasing inflammation and airway hyperreactivity. These effects depend in part on mitochondrial transfer and communication between cells. Consequently, iPSC-MSCs have the potential to treat COPD and other lung diseases caused by OS.

Finally, suppressing inflammation is one of the important ways to alleviate OS, and exosomes can also improve OS in other ways. For example, they can play a role through various means such as the NF-κB signaling pathway (81) and the Nrf2/Keap1 signaling pathway (82), and can also regulate mitochondrial membrane potential and reduce the production of mitochondrial ROS (83).

### 3.2 Wnt/β-catenin signaling pathway

The Wnt/β-catenin signaling pathway contains an abundance of glycoproteins with unique characteristics and is associated with a variety of physiological activities and diseases. It can participate in growth and development, physiological homeostasis, and tissue recondition (84). Many reports suggest that MSCs and their exosomes positively contribute to the treatment of the nervous system, cardiovascular system, skin, and other diseases through the Wnt/β-catenin signaling pathway.

Wnt signaling plays multiple roles in the neurogenesis, self-renewal, and homeostasis of neural stem/progenitor cells (NSCs) (85). Severe spinal cord injury (SCI) is currently incurable, and its pathological changes are complex. However, effective relief of SCI can be achieved through the improvement and recovery of neuronal function. Li et al. (22) designed a model of SCI, culturing neuron cells using exosomes derived from BMSCs (BMSC-Exos) at a concentration of 100mg/ml, and the data proved that BMSC-Exos could significantly reduce the protein expression levels of Bax, cleaved caspase-9, and cleaved caspase-3 and increase the expression of Bcl-2, β-catenin, and TCF-4. The results also indicated that the spinal cord and neurons were in a more mature state, and the number of neurons was greater. Their study elucidates the role of BMSC-Exos in reducing tissue damage, promoting repair, and inhibiting neuronal apoptosis by activating of Wnt/β-catenin signaling pathway. The occurrence of PD is related to the degeneration and death of dopamine neurons in the substantia nigra, and is the result of the combined action of multiple factors (86). Altarche-Xifro et al. (23) discovered that implantation of hematopoietic stem and progenitor cells into PD mouse models can fuse with neurons and some with glial cells, and then activate the Wnt/β-catenin canonical pathway, which prevents microglia from secreting pro-inflammatory cytokines. Therefore, the Wnt/β-catenin signal is crucial for maintaining the normal function of neurons and can provide help for the treatment of PD and AD (87). Sha et al. (24) emphasized that BMSC-EVs can be absorbed by neurons and released the miR-29c-3p carried by them, up-regulating the level of miR-29c-3p can inhibit BACE1, activate the Wnt/β-catenin pathway, decrease the expression of Aβ1-42 IL-1β, TNF-α, and IL-6, increase neuron viability and reduce apoptosis, thus it has a curative effect on the occurrence and development of AD. These results imply that the Wnt/β-catenin pathway is of great importance in brain development and the occurrence and development of nervous system diseases.

Ischemic stroke is caused by poor blood flow, leading to damage such as hypoxia and inflammation, resulting in cell death and apoptosis. Nazarinia et al. (25) Using 1cc of human amniotic mesenchymal stem cells (hAMSC-CM) in male Wistar rat model of middle cerebral artery occlusion to study cardiovascular diseases in the elderly; treatment with hAMSC-CM after cerebral reperfusion caused decreased infarct size, inhibition of inflammation, and apoptosis, enhanced antioxidant capacity, and increased activity of the Wnt/β-catenin signaling pathway. In addition, the degree of antioxidants is related to the form of FoxO1 protein. Reperfusion therapy for ischemic heart disease can cause myocardial cell injury, which can lead to heart failure when severe. Cui et al. (26) injecting 200µL PBS containing 400µg of exosomes derived from ADMSCs (ADMSC-Exos) into the tail vein of rats in the myocardial I/R model, found that ADMSC-Exos can significantly reduce I/R-induced cardiomyocyte apoptosis, up-regulate the levels of Bcl-2 and Cyclin D1, down-regulate the expression of Bax, and inhibit the activity of Caspase3. In addition, ADMSC-Exos promoted the activation of the Wnt/β-catenin signaling pathway through the regulation of Wnt3a, p-GSK-3β (Ser9), and β-catenin.

On the other hand, skin trauma is a soft tissue injury that is difficult to cure with age. The role of the Wnt pathway in skin wound healing cannot be ignored. Zhang et al. (27) revealed that exosomes derived from human umbilical cord MSCs (hucMSC-Exos) can enhance the nuclear transfer of β-catenin, increase the levels of N-cadherin, cyclin D3, and b-catenin, and decrease the levels of E-cadherin. These results indicate that hucMSC-Exos regulated endothelial cell proliferation, migration, vascular sprouting, remodeling, and vascular system maturation by releasing Wnt4 and activating the Wnt/β-catenin signaling pathway. During the aging process, skin injuries are difficult to heal. Ma et al. (28) prepared a skin lesion model by exposing HaCaT cells to hydrogen peroxide to reveal its possible mechanism of action. Their result indicated that HaCaT cells can be promoted to proliferate and migrate by ADMSC-Exos, meanwhile, ADMSC-Exos inhibit apoptosis and increase the expression of β-catenin to enhance the activation of the Wnt/β-catenin signaling pathway.

### 3.3 MAPK signaling pathway

Mitogen-activated protein kinases are involved in various cellular processes including embryogenesis, proliferation, apoptosis, and differentiation. The most common are ERK1/2, JNK(1–3), and p38(α, β, γ, and δ) families (88). MSCs are capable of treating age-related diseases through the MAPK signaling pathway under different conditions.

The various cellular damage caused by stroke is difficult to restore to the previous state of health. Song et al. (29) injected human dental pulp stem cells (hDPSCs) (4×10^6^ cells) into the caudal vein of the rat stroke model and ischemia model, *in-vivo* data confirmed that hDPSCs induce neuronal survival by releasing neuroprotective and anti-inflammatory molecules, various cytokines and growth factors through up-regulating the MAPK signaling pathway. Femoral head necrosis are caused by various reasons of insufficient blood supply, resulting in bone cell necrosis and changes in the structure, which may eventually lead to the collapse of the femoral head. Liao et al. (30) on osteonecrosis of the femoral head indicated that over-expression of miR-122-5p in exosomes increased the levels of VEGFR-1, PDGFR-α, ERK, JNK, and p38, reduce the expression of SPRY2 and enhanced the activity of receptor tyrosine kinase RTK, thus promoting osteoblast proliferation, differentiation, osteogenesis, and angiogenesis. OA is a degenerative change in articular cartilage that involves other structures such as synovium and joint capsule. It is common in the knee and hip joints (89). Ma et al. (31) concluded that TGF-β induced BMSCs can activate p38, and regulate the levels of sGAG, type II collagen, SOX9, and other genes by regulating P38/ERK/JNK and other signaling pathways, which has a guiding role in the treatment t of OA.

Treatments for other age-related diseases have also been reported. In terms of respiratory diseases, Gu et al. (32) showed that MSCs may reduce airway inflammation and emphysema in a rat model of cigarette smoke exposure, by inhibiting COX-2/PGE2 in alveolar macrophages, which is partly regulated by p38 MAPK and ERK signaling pathways; this is strong scientific evidence that MSCs alleviates COPD. In cardiovascular disease, Takafuji et al. (33) injected 10×MSC-CM 200μL twice a week into the tail vein of mice on a high-fat diet, and found that in TNF-α-stimulated human aortic endothelial cells, both the MSC EVs and conditioned medium from cultured MSCs (MSC-CM) supernatant could decrease the number of cell adhesion molecules by inhibiting MAPK pathways; similarly, in macrophages, MSC-CM also acts by inhibiting this signaling pathway, which may lessen the damage caused by coronary artery disease.

### 3.4 Sirtuin

The sirtuin (SIRT) family consisting of seven proteins (90), can participate in many biological metabolic pathways, including cell growth, proliferation, senescence, and apoptosis. Members of this family of enzymes have been identified to be ginvolved in human physiology and pathology processes and disease genesis, including cardiovascular diseases, neurodegenerative diseases, and so on (91). MSCs and their exosomes can also treat age-related diseases through this pathway, which has the potential for development despite related studies remaining incomplete.

In the treatment of myocardial infarction MI, Mao et al. (34) injected 40μg of MSC-Exos into each MI rat, and found that the lncRNA KLF3-AS1 in human MSC-Exos can inhibit cardiomyocyte activity, inflammation, and apoptosis by regulating the mir-138-5p/SIRT1 signaling pathway, thereby alleviating the symptoms of MI. The data showed that SIRT1 not only inhibited the expression of NLRP3 but also suppressed caspase-1 and inflammatory factors, suggesting that SIRT1 inhibits NLRP3 inflammasome activation and has a protective effect on vascular endothelial cells (92). Chae et al. (35) inserted the SIRT1 gene into a genomic locus in amniotic MSCs (AMMs) and investigated therapeutic potentials to treat a mouse model of collagen-induced arthritis (CIA): they discovered that AMMs transplantation blunted CIA progression inhibited Th-17 cell activation, and decreased the levels of pro-inflammatory factors, such IL-1β, TNF-α, MCP-1, and IL-6 while increasing the number of Treg cell in CIA mice which can help repair and treat damaged cartilage or inflammatory arthritis. Furthermore, as age increases, photoaging and photodamage are inevitable, and preventing or treating UV radiation can effectively alleviate it. Wu et al. (36) treated the acute skin damage model caused by UV radiation with 600µg of hucMSC-Exos, they believed that hucMSC-Exos could promote skin regeneration and repair, and studies found that it also can promote the expression of SIRT1 in HaCaT cells by transporting 14-3-3ζ protein and enhance autophagy activation to alleviate the OS injury induced by UV radiation and H_2_O_2_.

### 3.5 Exosomes derived from the SASP

Aging cells can secrete senescence-associated secretory phenotype (SASP), which is an important characteristic of cell aging. SASP includes interleukins, inflammatory factors, chemokines, proteases, and growth factors, among others. These factors can play various roles through autocrine or paracrine effects, such as participating in the aging, inflammation, and tumor formation processes (93). And there is research showing that aging may increase the secretion of exosomes (94). The functions of exosomes derived from SASP are also diverse. For example, exosomes derived from M2 macrophages can deactivate the TLR4/NF-κB/NLRP3 signaling pathway, increase cardiomyocyte vitality, inhibit inflammatory reactions, and reduce I/R injury (95). However, M1 macrophages release pro-inflammatory exosomes, which accelerate the damage of MI (96). Similarly, exosomes derived from dendritic cells can exacerbate atherosclerosis in mice (97), but can also have a protective effect on MI and I/R (98). From this, it can be seen that the functions of exosomes secreted by different cells vary, and the functions of exosomes also change with changes in the physiological state of the cells.

### 3.6 Clinical trials

We also summarized the relevant clinical trials conducted in recent years. Kim et al. treated 9 Alzheimer’s disease patients with intraventricular injection, with 3 patients receiving 1.0 × 10^7^ cells/2mL of huMSCs and 6 patients receiving 3.0 × 10^7^ cells/2mL of hUCB-MSC for long-term follow-up to explore therapeutic effects on AD (99). Although it can not show the clinical effect for the time being, it also provides a reference for future clinical research. Assia Jaillard et al. conducted a randomized controlled trial of ischemic stroke, they administered intravenous injections of BMSCs to patients with moderate to severe ischemic stroke occurring within 2 weeks, and found that exercise-related measures and scores improved after BMSCs treatment (100). Bartolucci et al. injected 1 × 10^6^ Cells/kg umbilical cord-derived MSCs (UC-MSCs) into patients with heart failure and decreased ejection fraction (101). There were no adverse events during the trial process, and the patients’ conditions and quality of life improved, indicating that intravenous UC-MSCs infusion is safe and feasible. Mathiasen et al. injected 0.2 mL of BMSCs intramyocardially 10-15 times in 60 patients with ischemic heart failure, and found cardiac systolic function was obviously improved (102). OA is common in elderly people, causing joint pain, deformity, and limited movement. In severe cases, surgical treatment is required to replace the joint. We have also summarized the related clinical studies on OA. For example, Chahal et al. injected different concentrations of BMSCs (1 × 10^6^ cells, 10 × 10^6^ cells and 50 × 10^6^ cells) into the knee joints of patients with knee OA. The final results showed a reduction in inflammatory cells, decreased levels of pro-inflammatory factors, and reduced pain in patients (103). Besides that, Emadedin et al. conducted a randomized controlled trial in which they implanted BMSCs (40 × 10^6^ cells) into the joints of knee OA patients. After 6 months of follow-up, they found improvements in pain and walking distance (104). Similarly, Matas and his colleagues’ clinical trial also involved injecting UC-MSCs (20 × 10^6^ cells) into the knee joint of patients with knee OA. What sets this trial apart from others is that it is a repeated injection trial within the joint (105). There have also been some exosome trials, but many have not yet been completed, with no published results available at the time of writing. The specific experimental content is summarized in **Table 2**. In summary, the majority of trials are conducted to verify the safety and effectiveness of clinical applications of stem cells and their exosomes. However, some trials may not have produced accurate conclusions due to limitations such as short follow-up time and small sample size. Therefore, more large-scale randomized controlled trials are needed to verify the efficacy of stem cells and their exosomes.

**Table 2:** Clinical trials of MSCs and their exosomes in the treatment of age-related diseases.

Source of MSCs/Exos	Disease	Route	Dosage/cells	Follow-up period	Participants	Benefits	Adverse events	Ref./Number
UC-MSCs	AD	Intracerebroventricular injections	1.0 × 10^7^ cells 3.0 × 10^7^ cells	36 months	9	As this was an open-label phase I clinical trial, we could not prove the clinical efficacy of hUCB-MSC injection	The most common adverse event was fever, headache, nausea, and vomiting	(99)
BMSCs	Ischemic Stroke	Intravenous injection	1.0×10^7^ 3.0×10^7^	2 years	31	Improve motor recovery through sensorimotor neuroplasticity	Seizures, urinary tract infection, algodystrophia, pneumonia, and so on	(100)
UC-MSCs	Heart failure	Intravenous Infusion	1×10^6^ cells/kg	12months	30	Left ventricular function, functional status and quality of life were significantly improved	Stroke, sustained ventricular arrhythmias, incident malignancy, and so on	(101)
BMSCs	Ischaemic heart failure	Intramyocardial injections	0.2 mL	12months	60	Improve myocardial function and myocardial mass in patients	Double-vision, dizziness, stroke, angina, and so on	(102)
BMSCs	Osteoarthritis	Intra-articular injection	1 × 10^6^ 10 × 10^6^ 50 × 10^6^	24 months	12	Symptoms of osteoarthritis improve, with less pain and stiffness	Local transient adverse events such as pain and/or swelling at the injection site	(103)
BMSCs	Knee os-teoarthri-tis	Intra-articular implantations	40×10^6^cells	6 months	43	WOMAC total score, pain, and painless walking distance improved significantly	There were no major adverse events	(104)
UC-MSCs	Knee osteoarthritis	Intra-articular injection	20 × 10^6^	1 year	26	Pain and function improved	The most common adverse events were acute synovitis and pain	(105)
MSCs-Exos	Ischemic Stroke	Intraparanchymal injection		12months	5			NCT03384433
MSCs-Exos	AD	Nasal drip	5μg/ml 10μg/ml 20μg/ml	48 weeks	9			NCT04388982
MSCs-Exos	Knee osteoarthritis	Intra-articular Injection	3-5×10^11^	12months	10			NCT05060107

UC-MSCs, umbilical cord-derived MSCs; AD, Alzheimer’s disease; BMSCs, bone marrow-derived MSCs; MSCs-Exos, exosomes derived from MSCs.

### 3.7 Summary

From the above discussion, we can learn that MSCs and their exosomes can alleviate age-related diseases in multiple systems through these four signaling pathways.

**Figure 2:**
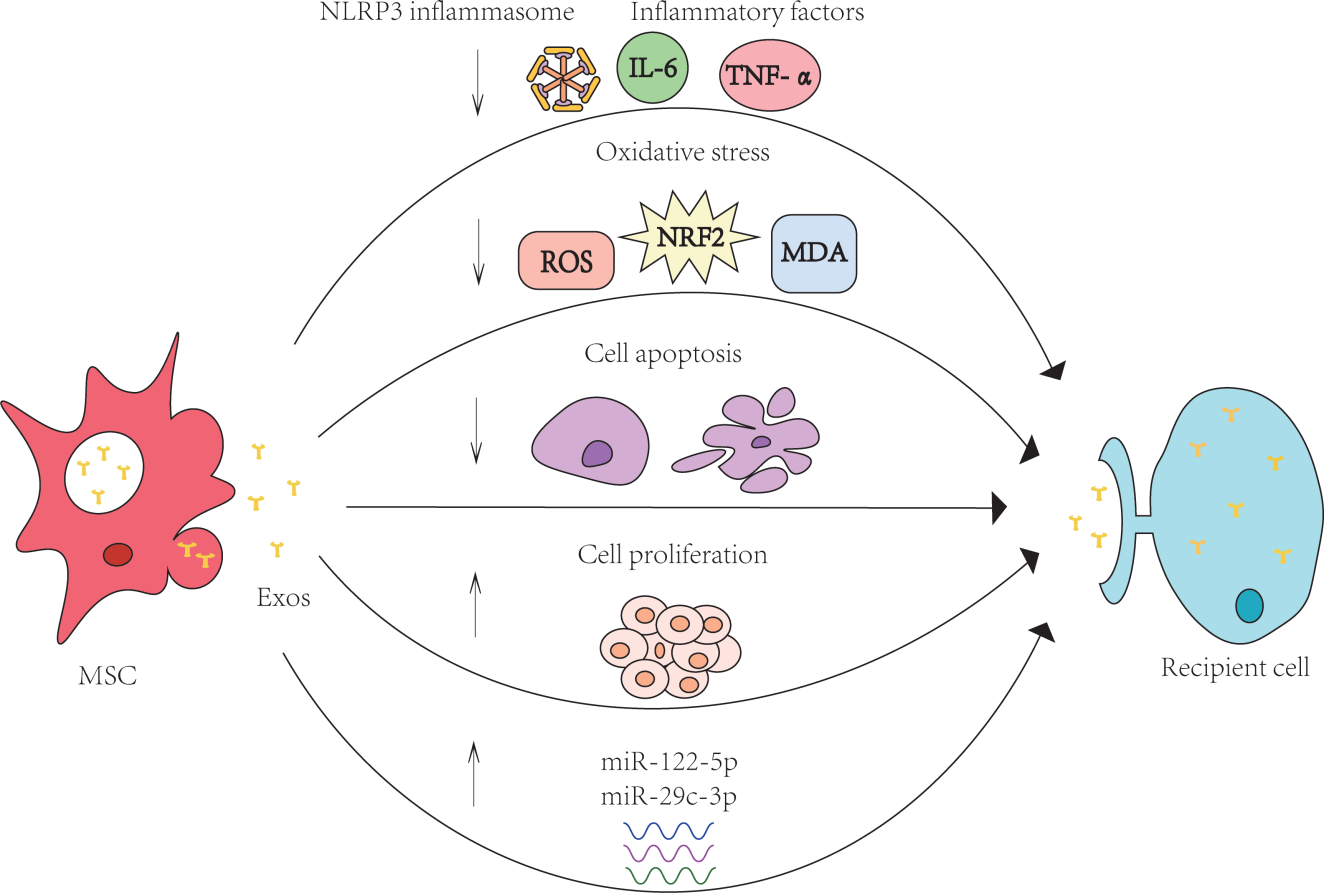
Specific mechanisms of MSCs and their exosomes in the treatment of age-related diseases. The specific mechanisms by which exosomes exert their effects include anti-inflammatory, antioxidant, anti-apoptotic, promotion of cell proliferation, and serving as carriers to transport various substances, and so on.MSC: mesenchymal stem cell; Exos: exosomes.

The specific mechanisms by which exosomes exert their effects include anti-inflammatory, antioxidant, anti-apoptotic, promotion of cell proliferation, and serving as carriers to transport various substances, and so on. (**Figure 2**)

## 4 Problems and prospects

Many authors show support for the use of MSCs and their exosomes which can, through various mechanisms, be used to treat age-related diseases, but these mechanisms are complex, and there may be cross-over and synthesis issues arising therewith. In OS mechanisms, MSCs are able to inhibit the expression of Nox2 in the skin caused by I/R, however, the current problem is that Nox2 is mainly expressed in macrophages and neutrophils, and also in vascular endothelial cells and fibroblasts (106), thus, it has not been thoroughly explored as to which cells MSCs inhibit to exert their regulatory effects on OS. In the Wnt/β-catenin signaling pathway, although studies clarified that hucMSC-Exos may achieve a therapeutic effect on skin by promoting angiogenesis (27), it is unclear which of these components are involved in promoting angiogenesis. There is also evidence that ADMSC-Exos could alleviate I/R-induced myocardial injury through the Wnt/β-catenin signaling pathway (26). Nevertheless, the present study does not expound how ADMSC-Exos regulate Wnt/β-catenin signaling and which molecules secreted by ADMSC-Exos are engaged in the regulation. At present, the evidence is not comprehensive, and more detailed mechanisms of action should be investigated.

In addition, when it comes to a specific pathway, the mode of action of this pathway is changed. MSCs and their exosomes can activate this pathway to play a positive role and inhibit this pathway under certain conditions. For example, in the MAPK signaling pathway, MSC-Exos promote the expression of related proteins therein, thus promoting osteoblast proliferation and improving osteoporosis (107). miR-181a-2-3p encapsulated in extracellular vesicles derived from MSCs can regulate EGR1 and inhibit apoptosis and MDA and ROS levels by inhibiting NOX4/p38 MAPK, thereby reducing OS in PD (108). Likewise, the expression of Wnt/β-catenin signaling is different in various diseases: for example, Wnt/β-catenin signaling is up-regulated in bipolar disorder but down-regulated in AD, PD, and other diseases, making it more difficult to develop novel therapeutic strategies. It is also important to note whether *in vitro* and *in vivo* studies of the same mechanism have been conducted and whether their conclusions are consistent.

Finally, in clinical trials, exosomes can be used not only as biomarkers, but also as a vehicle with stronger targeting ability and lower cytotoxicity. Multiple preclinical data have shown that in disease treatment, exosomes may have better safety and functionality than cellular therapy (109). However, in clinical experiments, the use of exosomes also has some drawbacks. The activity of the loaded substances may be lost in the process of delivery. The specific effect of exosomes should also consider the cell source, isolation and storage, dose and other issues. Complete production equipment and quality assurance are the most important factors for the mass production of exosomes (110), because different production processes will cause the function of exosomes to be different, thus causing interference to the treatment effect of diseases (111). Firstly, according to the MISEV2018, complete purification of exosomes is almost impossible. Instead, multiple purification methods should be combined according to the specific use of EVs. According to the recovery and specificity of EVs, the guidelines give four recommendations, one of which should be selected for the application of a specific isolation technique. If there are special circumstances, they should also be stated (112). Secondly, thrombosis and hemostatic disorders may be a difficult problem for the widespread application of exosomes in systemic therapy. Notably, the larger the exosome, the more likely it is to lead to the production of pro-thrombotic factors (113). Thirdly, the most important thing is to establish uniform potency criteria for exosomes, which is a key step toward clinical application (114). Finally, problems of separation and storage need to be solved, and more importantly, production is required to be perfected with technology that can guarantee patient safety (115).

## 5 Conclusion

In this review, we summarized the various mechanisms of MSCs and their exosomes in the treatment of age-related diseases, including OS, Wnt/β-catenin signaling pathway, MAPK signaling pathway, and SIRT family. At present, research into the therapeutic effects of MSCs and exosomes on aging diseases remains in the preclinical stage, which warrants further clinical trials, but much of the literature shows that the direction of study can provide new treatment methods and strategies for clinical application.

## Author contributions

LY and HS contributed to the conception of this manuscript. JL and YH drafted and revised the manuscript. All authors contributed to the article and approved the submitted version.

## Glossary

**Table:**

MSCs	mesenchymal stem cells
MSC-Exos	exosomes derived from MSCs
OS	oxidative stress
MAPK	mitogen-activated protein kinases
I/R	ischemia-reperfusion
IVDD	intervertebral disc degeneration
PD	Parkinson’s disease
iPSCs	pluripotent stem cells
ESCs	embryonic stem cells
BMSCs	bone marrow-derived mesenchymal stem cells
ADMSCs	adipose-derived MSCs
huMSCs	human umbilical cord-derived MSCs
hpMSCs	human placenta-derived MSCs
PDGF	platelet-derived growth factor
VEGF	vascular endothelial growth factor
IL-1	interleukin-1
TGF	transforming growth factor
MSC EVs	mesenchymal stem cell-derived extracellular vesicles
PM	plasma membrane
EEs	early endosomes
MVBs	multivesicular bodies
ILVs	intraluminal vesicles
mRNA	messenger RNA
miRNAs	microRNAs
TGN	trans Golgi network
AD	Alzheimer’s disease
UV	ultraviolet
COPD	chronic obstructive pulmonary disease
AβOs	amyloid-β peptide oligomers
LDL	low-density lipoprotein
DBMSCs	decidua basalis mesenchymal stem/multipotent stromal cells
ROS	reactive oxygen species
iPSC-MSCs	induced-pluripotent stem cell-derived MSCs
ASMCs	airway smooth muscle cells
SCI	spinal cord injury
BMSC-Exos	exosomes derived from BMSCs
hAMSC-CM	human amniotic mesenchymal stem cells conditioned medium
ADMSC-Exos	exosomes derived from ADMSCs
hucMSC-Exos	exosomes derived from human umbilical cord MSCs
hDPSCs	human dental pulp stem cells
OA	osteoarthritis
MSC-VEGF-CM	MSCs over-expressing VEGF conditioned medium
MSC-CM	conditioned medium from cultured MSCs
MI	myocardial infarction
AMMs	amniotic MSCs
CIA	collagen-induced arthritis
SASP	senescence-associated secretory phenotype
UC-MSCs	umbilical cord-derived MSCs
